# Benzathine Penicillin G Shortage and Secondary Syphilis

**DOI:** 10.7759/cureus.66787

**Published:** 2024-08-13

**Authors:** William Snider, Ian Depew, Shane Cook, Danielle Roth

**Affiliations:** 1 Dermatology, Marshall University Joan C. Edwards School of Medicine, Huntington, USA

**Keywords:** bpg shortage, nonspecific skin eruptions, secondary syphilis, doxycycline, sexually transmitted disease, syphilis

## Abstract

Syphilis is a sexually transmitted infection caused by the bacterium *Treponema pallidum*. This disease is characterized by four different stages, each presenting with a variety of manifestations or asymptomatic disease. These stages can be further broken down into early-stage syphilis, which includes primary and secondary syphilis, and late-stage syphilis, which includes tertiary syphilis. It is crucial to recognize and treat syphilis early because the later stages of the disease are marked by irreversible damage to the central nervous system (CNS) and cardiovascular system, and can even increase mortality risk. The primary recommended treatment for early-stage syphilis is intramuscular (IM) benzathine penicillin G (BPG). In this case report, we present a patient with secondary syphilis who exhibited red papules and nonspecific skin eruptions. Due to the unavailability of BPG, the patient initially received doxycycline as an alternative treatment. After eight days of searching multiple facilities and pharmacies, a dose of BPG was finally located and administered to the patient. We highlight crucial information about the BPG shortage, including supply and demand challenges, infrastructure issues, and the broader impact on numerous other antimicrobials. We emphasize the importance of recognizing this issue and provide alternatives for managing the disease in resource-limited settings.

## Introduction

Syphilis, classified as a sexually transmitted infection caused by the bacterium *Treponema pallidum*, progresses through four distinct stages: primary, secondary, latent, and tertiary. With further classification, primary and secondary syphilis are categorized as early syphilis, while tertiary syphilis is classified as late syphilis. Each stage is marked by unique clinical manifestations. Owing to its diverse presentation, syphilis is renowned as a remarkable imitator of various conditions [1]. A comprehensive diagnostic approach is warranted for individuals displaying genital ulcers, symmetrical and diffuse skin eruptions, as well as neurological symptoms without an identified cause [1].

Syphilis has seen a dramatic resurgence, with primary and secondary cases increasing. In 2020, there were 7.1 million new adult cases worldwide [2]. Contributing factors include unsafe sexual practices, reduced screening, healthcare worker shortages, and the discontinuation of STI (sexually transmitted infection) programs during COVID-19 [3].

Diagnosis is crucial for symptomatic and high-risk individuals, and IM benzathine penicillin G (BPG) stands as the primary treatment for early syphilis and late-stage syphilis [4]. While BPG is inexpensive to purchase, it is very costly to manufacture [5]. BPG is also used as the primary treatment for secondary prophylaxis of rheumatic heart disease, the primary treatment for group A streptococcal pharyngitis, and yaws [6]. This, combined with limited suppliers, complex manufacturing, and inaccurate syphilis rate forecasting, has led to BPG shortages and increased use of alternative treatments like doxycycline, azithromycin, and ceftriaxone, each with drawbacks [5-9]. Past studies highlight the need for improved BPG supply chains; however, many do not go into the supply and demand factors that contribute to these shortages [5]. In this case, we present a patient with secondary syphilis who exhibited red papules and nonspecific skin eruptions. Since IM BPG was not immediately available, the patient initially received doxycycline as a secondary treatment for eight days until IM BPG could be located and administered. This case report delves into the shortage issue, supply and demand chain problems, and external factors contributing to the shortage. The current case was previously presented as a poster at the 2023 West Virginia Dermatological Society Meeting on August 11, 2023.

## Case presentation

**Figure 1:**
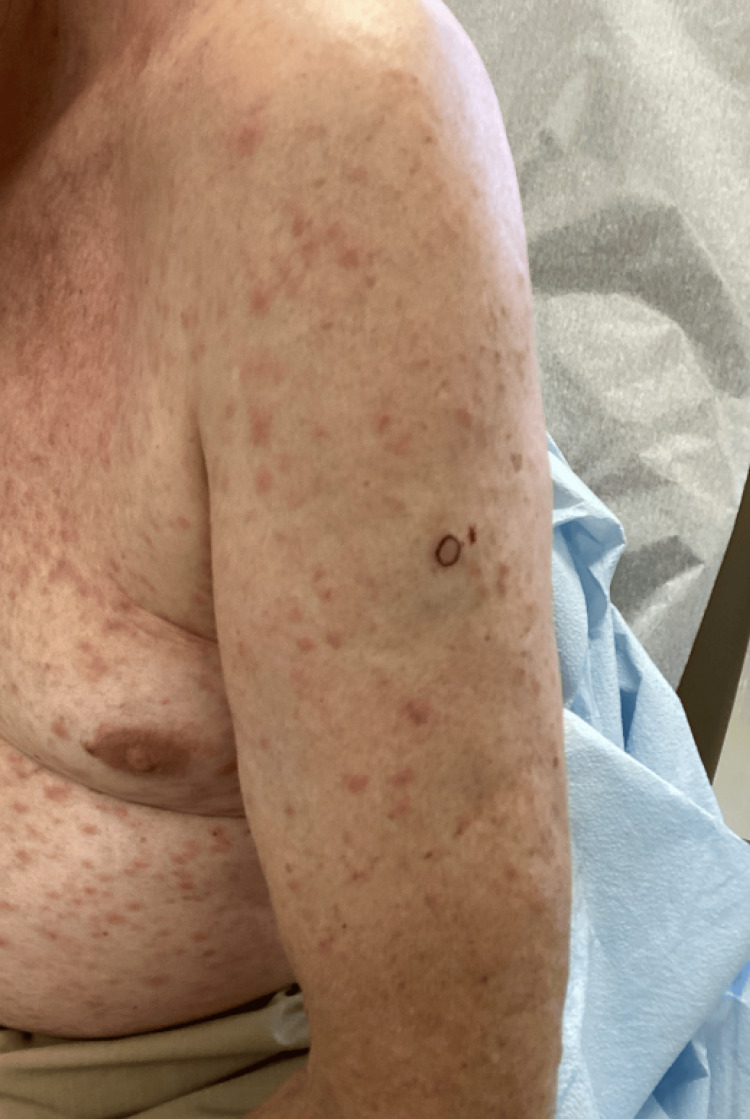
Location of punch biopsy on left arm demonstrating the non-pruritic red papules distributed across the arms, chest, and legs.

A 71-year-old male presented to the dermatology outpatient clinic with a widespread skin eruption that began one year ago. The patient did not seek treatment, as he believed it was related to an artificial sweetener allergy. His medical history was unremarkable. On physical examination, the skin eruption consisted of non-pruritic red papules distributed across his arms, chest, and legs (Figure 1). Notably, his palms and soles were unaffected.

**Figure 2:**
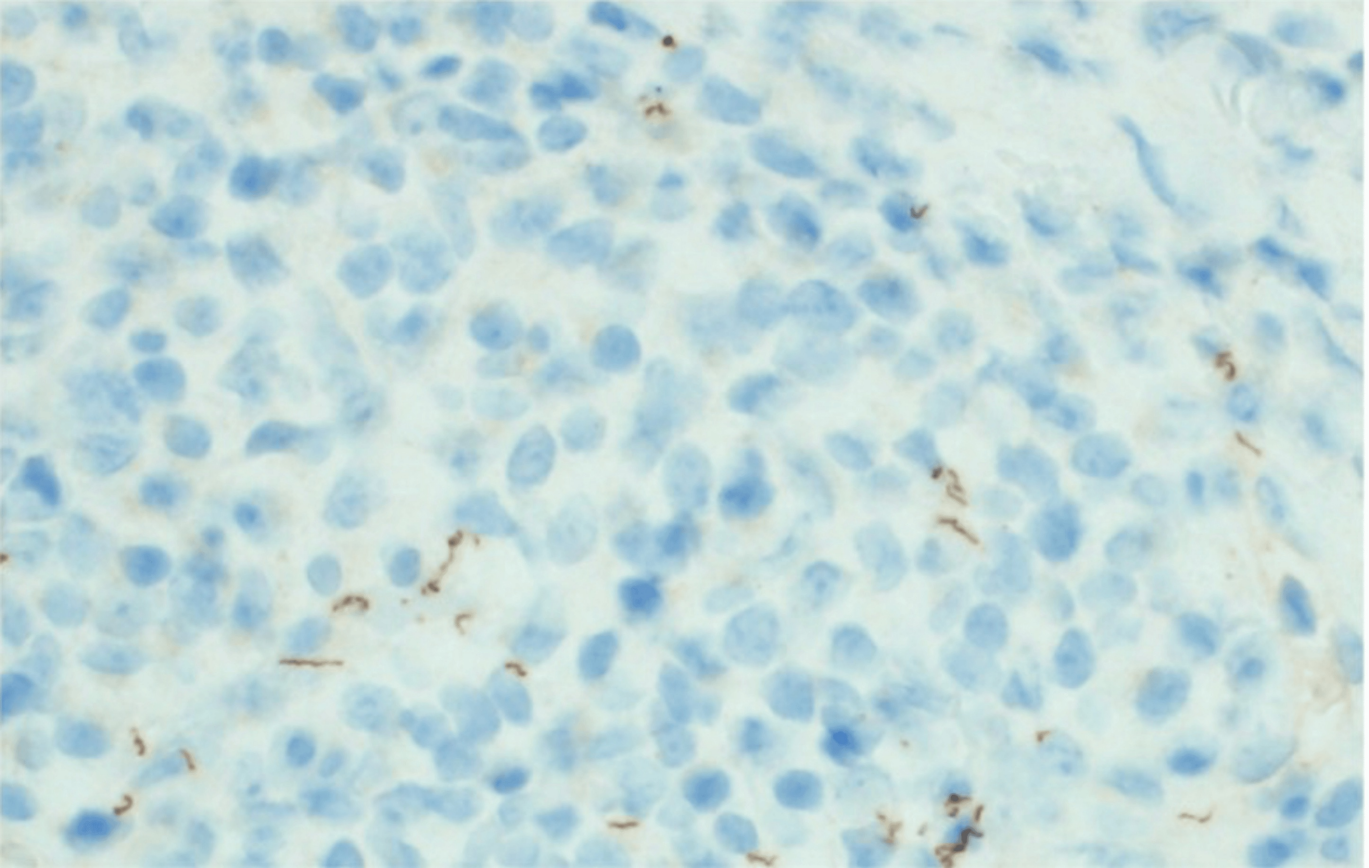
Treponema pallidum Immunostaining (400x).

A punch biopsy was performed, revealing a lichenoid interface dermatitis characterized by delicate rete ridges. A spirochete immunostain highlighted numerous spherocytes within and around the dermal vessels (Figure 2). Following this finding, the patient was found to have a reactive *Treponema pallidum* antibody (fluorescent treponemal antibody absorption (FTA-ABS)) test, leading to a diagnosis of secondary syphilis.

The initial treatment plan involved administering BPG. However, despite searching through our clinic and neighboring primary care facilities, BPG was unavailable. We contacted the patient's primary care physician and several local pharmacies but were unable to secure BPG. Consequently, the patient received a 14-day course of doxycycline. Eight days post-diagnosis, a supply of BPG was located at the infectious disease specialist clinic, and the patient received intramuscular penicillin G. Before treatment, his rapid plasma reagin (RPR) test was 1:256. Follow-up RPR tests at 6 and 12 months showed levels reduced to 1:4 with resolution of the skin rash. The infectious disease team plans to continue monitoring RPR levels every six months.

## Discussion

Syphilis, a sexually transmitted infection caused by the bacterium *Treponema pallidum*, has experienced a significant resurgence in recent years. According to a review of CDC (Centers for Disease Control and Prevention) data, there has been a staggering 505% increase in primary and secondary syphilis cases since 2000, along with a 301% rise in congenital syphilis cases [7]. According to the World Health Organization, in 2020, there were 7.1 million new cases worldwide among adults [2]. This underscores the critical importance for healthcare providers to remain aware of this alarming trend and to comprehend the evolving landscape of STI prevalence.

The upsurge in syphilis cases can be linked to several contributing factors, including unsafe sexual practices, decreased efforts in screening, a shortage of healthcare personnel, and the discontinuation of 84% of programs dedicated to addressing sexually transmitted infections during COVID-19 [3]. When suspicion of syphilis arises, a thorough diagnostic approach is crucial for individuals presenting with genital ulcers, symmetrical and diffuse skin eruptions, as well as general paresis. Additionally, asymptomatic individuals at heightened risk due to engaging in risky sexual behaviors, particularly men who have sex with men, those with compromised immune systems, and individuals with substance abuse issues, require diligent screening efforts [8].

Upon a positive diagnosis of early-stage syphilis, as seen in this case, IM benzathine penicillin G (BPG) remains the primary treatment of choice (Table 1) [4]. This is because syphilis has not developed resistance to this first-line treatment [5]. When BPG is unavailable, several alternative treatments are available, although each comes with drawbacks. The first alternative is doxycycline, taken orally twice daily for 14 days. However, doxycycline is not recommended for use during pregnancy or in young children. Second, oral azithromycin has been effective in treating early syphilis, but growing resistance has limited its usage. Lastly, ceftriaxone requires daily IM/IV injections for 10-14 days, which is often impractical in the clinical setting. Due to limited data on the efficacy and safety of these alternative regimens, pregnant women with a penicillin allergy must first undergo penicillin desensitization [2]. Table 1 highlights the top two antibiotic options for treating early syphilis.

**Table 1:** Comparison of BPG vs. doxycycline treatments for early-stage syphilis [4].

Benzathine Penicillin G (BPG)	Doxycycline
Single dose (2.4 million units IM once)	100 mg twice daily for 14 days
Safe in pregnancy	Avoid in pregnancy
Microbe’s slow growth rate makes penicillin’s prolonged exposure recommended.	Close monitoring required

When considering the reasons behind the BPG shortage from a top-down perspective, it is clear that this is a multifaceted issue involving numerous factors. A comprehensive survey conducted in 2017 that assessed BPG shortages and stock-outs across 114 countries during 2014-2016 highlighted a notable trend: out of the 95 countries that responded to the surveys, 39 (41%) faced shortages of BPG [5]. Uniquely, the study found a supply chain shortage of Pfizer’s BPG product in five high-income countries (the United States, Canada, New Zealand, the Netherlands, and Germany). It also found that larger wholesale agents, such as the United Nations Children’s Fund (UNICEF), were experiencing challenges sourcing BPG for buyers [5]. When it comes to producing BPG, it costs US$0.11 for a 1.2 million IU dose and US$0.20 for a 2.4 million IU dose. The investment includes the sterile injection infrastructure required to produce one unit of BPG. This involves both an active pharmaceutical ingredient (API) manufacturer, who produces the active product of BPG, and a final dose formulator (FDF) manufacturer, who formulates, packages, and labels the BPG. This infrastructure requirement, combined with high infrastructure costs and low market returns, has resulted in limited new market entrants and contributed to 40 FDFs and six APIs exiting the market by the early 2000s [5]. While API manufacturers can meet global demands, they optimize sales by employing a constrained supply chain. This means they set minimum purchase orders, which create challenges for countries that do not meet the minimum number to order BPG units [5]. Furthermore, API manufacturing quality is an increasing issue, where certain API manufacturers are not meeting strict regulatory authority standards for BPG quality. Likewise, API manufacturers produce slightly different technical specifications (e.g., particle size, excipient specifications) for certain FDF manufacturers, meaning that if one API manufacturer were to go offline, this would cause a delay for the FDF manufacturer.

On the demand side, the investigation identified key trends: the challenge of accurately forecasting syphilis rates, inflexible purchasing cycles using historical BPG consumption data instead of facility-level data due to the unavailability of that data, and limited BPG product registrations when a country relies on a single approved BPG supplier, limiting flexibility if that single supplier were to shut down or halt production [5]. These demand issues highlight areas where policies should be improved for in-country purchasing and purchasing strategies [6]. This creates a demand problem independent of the supply chain issue.

Furthermore, in 2013, the World Health Organization developed a list of essential antimicrobials, including BPG, that satisfy basic requirements for their population's needs. This created a market cap where manufacturers could not charge more than a certain amount for essential medications. The unintended consequence was that certain antimicrobials were threatened as pharmaceutical companies determined they were inadequate to sustain manufacturing [9].

This issue extends beyond the BPG realm and includes numerous antimicrobials, specifically cefazolin, clofazimine, dapsone, rifabutin, cloxacillin, piperacillin-tazobactam, and ceftolozane-tazobactam [9]. The reasons behind these antimicrobial shortages are similar to the findings stated previously, in that scarcity arises from the inherent lack of profitability in the medication's production. BPG is sold for pennies but requires a large investment to produce the product. This is compounded by a fragmented production process, emphasizing the API and FDF manufacturing issues. Many APIs for antimicrobials are located in India and China, while FDFs are widely distributed. This means that any event causing an API to stall manufacturing causes the entire process to halt [9].

To further confound the issue, a study conducted in China's Shandong Province sheds light on an additional facet of this problem. China boasts three major API companies responsible for manufacturing the active ingredients required for BPG. Interestingly, while China itself does not grapple with BPG shortages, the study delves into the conundrum surrounding physicians' preference for BPG. It uncovers a blend of factors, including a lack of confidence in diagnosing the disease, the high incidence of penicillin allergies, and the prevalence of alternative treatment options. Together, these factors steer physicians away from BPG [10].

## Conclusions

This case highlights the critical importance for primary care facilities to not only understand the shortage and associated risks of BPG but also to take proactive steps to ensure its availability for immediate use in the clinical setting. This includes knowing where to refer patients when BPG is not available in the clinic and working with local pharmacies to ensure BPG is readily available and can be administered if needed. It is equally important to recognize that other key medications, such as cefazolin, clofazimine, dapsone, rifabutin, cloxacillin, piperacillin-tazobactam, and ceftolozane-tazobactam, may also experience similar shortages. In these cases, knowing how to access the medication and understanding the reasons behind the shortage is crucial. 

## References

[1] Peeling RW, Hook EW 3rd (2006). The pathogenesis of syphilis: the Great Mimicker, revisited. J Pathol.

[2] Stafylis C, Klausner JD (2024). Repurposing antibiotics to treat syphilis. Lancet Infect Dis.

[3] Nazir A, Masood W, Ahmad S (2022). Rise of syphilis surge amidst COVID-19 pandemic in the USA: a neglected concern. Ann Med Surg (Lond).

[4] Clement ME, Okeke NL, Hicks CB (2014). Treatment of syphilis: a systematic review. JAMA.

[5] Nurse-Findlay S, Taylor MM, Savage M (2017). Shortages of benzathine penicillin for prevention of mother-to-child transmission of syphilis: an evaluation from multi-country surveys and stakeholder interviews. PLoS Med.

[6] Wyber R (2021). Global status of BPG report. RHD Action.

[7] Black Black, M. L. (2023, February 6 (2024). STD rates spike by as much as 505% nationally since 2000; North Dakota and Utah see the biggest jumps. STD Rates Spike By As Much As 505% Nationally Since 2000; North Dakota and Utah See.

[8] Schmidt R, Carson PJ, Jansen RJ (2019). Resurgence of syphilis in the United States: an assessment of contributing factors. Infect Dis (Auckl).

[9] Shafiq N, Pandey AK, Malhotra S (2021). Shortage of essential antimicrobials: a major challenge to global health security. BMJ Glob Health.

[10] Chen X, Li G, Gan Y, Chu T, Liu D (2019). Availability of benzathine penicillin G for syphilis treatment in Shandong Province, Eastern China. BMC Health Serv Res.

